# A novel 25-ferroptosis-related gene signature for the prognosis of gliomas

**DOI:** 10.3389/fonc.2023.1128278

**Published:** 2023-04-20

**Authors:** Xiaojiang Peng, Chun Liu, Jing Li, Zeqing Bao, Tao Huang, Lingfeng Zeng, Qixiong He, Daojin Xue

**Affiliations:** ^1^ Zhaoqing Medical College, Zhaoqing, China; ^2^ Department of Neurosurgery, Lianshui People’s Hospital Affiliated to Kangda College of Nanjing Medical University, Huaian, China; ^3^ Department of Neurosurgery, The First Affiliated Hospital of Chongqing Medical University, Chongqing, China; ^4^ Department of Neurosurgery, The Second Affiliated Hospital of Guangzhou University of Chinese Medicine, Guangdong Provincial Hospital of Chinese Medicine, Guangzhou, China; ^5^ Department of Medicine, Chinese University of Hong Kong, Hong Kong, Hong Kong SAR, China

**Keywords:** ferroptosis, gene signature, prognosis, gliomas, bioinformatic

## Abstract

**Background:**

Ferroptosis is closely associated with cancer and is of great importance in the immune evasion of cancer. However, the relationship between ferroptosis and glioma is unclear.

**Methods:**

We downloaded the expression profiles and clinical data of glioma from the GlioVis database and obtained the expression profiles of ferroptosis genes. A ferroptosis-related gene signature was developed for the prognosis of gliomas.

**Results:**

We screened out prognostic ferroptosis genes, named ferroptosis-related genes, by the Cox regression method. Based on these genes, we used unsupervised clustering to obtain two different clusters; the principal component analysis algorithm was applied to determine the gene score of each patient, and then all the patients were classified into two subgroups. Results showed that there exist obvious differences in survival between different clusters and different gene score subgroups. The prognostic model constructed by the 25 ferroptosis-related genes was then evaluated to predict the clinicopathological features of immune activity in gliomas.

**Conclusion:**

The ferroptosis-related genes play an important role in the malignant process of gliomas, potentially contributing to the development of prognostic stratification and treatment strategies.

## Introduction

Glioma (GBM) is a kind of brain tumor, with an incidence of 5–6 cases per 100,000 people (1–3). Malignant glioma is a kind of highly invasive tumor, accounting for approximately 55% of gliomas, with a median survival period of 14-16 months (4, 5). Glioma has strong heterogeneity, which always leads to an increased recurrence rate after curative treatment (6). Despite aggressive treatments, the prognosis of malignant glioma is still poor. Hence, it is urgent to develop new therapeutic methods to enhance the prognosis of patients with glioma. Many scholars found that tumor cells have characteristics different from the cells of normal tissue, such as the avoidance of cell death (7, 8). The tumor microenvironment (TME) is considered a crucial factor in regulating invasion and preventing the destruction and survival of blood vessels (9–11). Glioma cells utilize their TME to maintain cellular homeostasis to avoid or reduce cell death (12). Regulated cell death (RCD) refers to death associated with gene regulation originating from the extracellular microenvironment which is essential to restore cellular homeostasis (13).

Ferroptosis, a newly discovered non-apoptotic RCD (14), is mediated by the accumulation of reactive oxygen species (ROS). In essence, under the action of ferroptosis, the accumulation of lipid peroxides leads to an imbalance in intracellular redox, leading to ferroptosis (15). Inhibition of glutathione peroxidase 4 (GPX4) or glutathione synthesis by small molecular compounds has been shown to result in ferroptosis (16, 17). Ferroptosis is associated with various diseases, such as ischemia–reperfusion injury, neurodegenerative diseases, and tumors. Several studies have shown that ferroptosis is a key factor in acquired drug resistance and immune evasion of cancer (18). In glioma, autophagy inhibition can improve the sensitivity of glioma stem cells to temozolomide (TMZ) by causing ferroptosis (19, 20). However, there are few reports on how ferroptosis occurs, develops, affects, and regulates glioma. It is important to study the biological significance of ferroptosis-related genes in glioma so as to construct a novel treatment method.

Recently, many researchers have been inclined to use bioinformatics methods to study the genesis and development mechanism of tumors. Using bioinformatics methods, we can determine the most appropriate samples for testing and identify the pathways and genes that can affect the development of glioma more quickly and accurately, thus providing more ideas for the treatment of glioma and the prediction of patient’s prognosis.

## Materials and methods

### Data source

Gene expression data and the glioma patient survival information in The Cancer Genome Atlas (TCGA) program were downloaded from the GlioVis GBM platform (http://gliovis.bioinfo.cnio.es/) (21), in which the RNA-seq data were processed by the normalized read counts from the preprocessed data with log2 transformation.

### Prognostic ferroptosis gene identification and prediction and PCA score calculation

**Figure 1:**
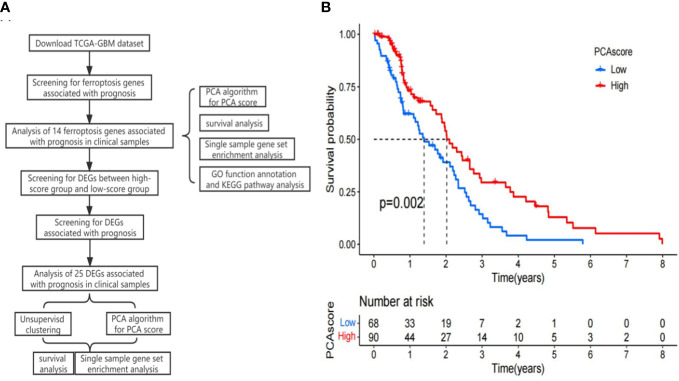
**(A)** Research flow chart. **(B)** Kaplan–Meier curves of the principal component analysis (PCA) scores in the TCGA-GBM.

We downloaded 253 ferroptosis regulators, 111 ferroptosis markers, and 95 ferroptosis–disease associations from FerrDb (http://www.zhounan.org/ferrdb). FerrDb can be used to obtain insights into ferroptosis (**Figure 1A**) (22). The ferroptosis gene expression data in the TCGA-GBM were obtained, and the ferroptosis genes significantly correlated with prognosis were determined based on the Cox regression method (*p*-value < 0.05). The expression matrix of relevant iron metabolism genes was extracted. Then, the score of each TCGA-GBM sample was calculated according to the principal component analysis (PCA) algorithm. PCA was applied to establish a ferroptosis-relevant score model, namely, the PCA score, and the PCA score was determined based on the formula: PCA score = (PC1*i* + PC2*i*), in which *i* represents the level of ferroptosis family pattern-related signature genes. Both PC1 and PC2 were regarded as signature scores. According to the threshold, the patients were classified into high- and low-score groups, while the relevant survival curves were generated and checked by the log-rank test between the two groups.

### Identification of differentially expressed ferroptosis-related genes

We identified the differentially expressed genes (DEGs) between the high-score and low-score groups. The “limma” package (23) of R was used to identify DEGs. Fold-change (FC) >1 and adjusted *p*-value <0.05 were set as the judgment standard for DEGs. An adjusted *p*-value <0.05 was applied to control the false-positive rate (24).

### Identification of the ferroptosis-related gene cluster in the TCGA-GBM

Based on the screened DEGs, unsupervised clustering was applied to cluster distant genotypes of the TCGA-GBM samples by the “ConsensusClusterPlus” package (25). Heatmaps were drawn to visualize the clinical characteristics of different gene clusters. The GBM score of each individual was also determined by these ferroptosis-associated DEGs, which we called the PCA gene score. In terms of cutoff value, the cases were classified into the high-gene score and low-gene score groups. Kaplan–Meier (KM) curves were generated and compared with the log-rank test between the high-gene score and low-gene score groups.

### Functional enrichment analysis

Gene ontology (GO) analysis (26) was used to analyze the function of the gene products and to identify their biological feature. The Kyoto Encyclopedia of Genes and Genomes (KEGG) (27) enrichment analysis was mainly used to explain gene function and was widely used in the bioinformatics research field. Based on DEG, it was mainly applied to the “ClusterProfiler” software (28) during the GO and KEGG analyses. The “gsva” package (29) was mainly used for single-sample gene set enrichment analysis (ssGSEA) to determine the proportion of infiltrating immune cells and also to determine the activity difference of immune pathways in different groups. The correlations of infiltrating immune cells and PCA score/gene score were shown in correlation heatmaps.

### Western blotting

The milled tissue and cells were lysed and boiled in sodium dodecyl sulfate (SDS) sample loading buffer, separated by 10% SDS-polyacrylamide gel electrophoresis (SDS-PAGE) and transferred to PVDF membranes. The membranes were blocked in 5% skimmed milk for 1 h, washed three times with TBST, incubated with the appropriate primary antibody in TBST for 1 h, then washed twice, and incubated with horseradish peroxide-conjugated anti-immunoglobulin (1:5,000 dilution) for 1 h at room temperature. After three washes with TBST, the membranes were developed using ECL-enhanced chemiluminescence reagents.

### Immunohistochemistry

Tissues were fixed in 4% paraformaldehyde for 24 h, followed by embedding in paraffin. Sections were cut to a thickness of 5 μm and incubated with anti-MAP1LC3A, anti-OLFM1, anti-CEND1, anti-CLTB, anti-PRKAR1B, anti-HRAS, anti-DDRGK1, anti-PITHD1, and anti-DEAF1 primary antibodies. Hematoxylin restaining was followed by separate photographic observation of each slide in a positive area of ×40 fields, which were independently analyzed by three previously uninformed pathologists.

### Statistical analysis

To compare the overall survival (OS) of the subgroups, the KM curve with the log-rank test was applied (30). In order to determine the prognostic value of the iron production death gene in the research process, this paper conducted Cox regression analysis on the collected data and applied the Mann–Whitney method when comparing the degree of immune cell infiltration of the two groups. The correlations of infiltrating immune cells and PCA score/gene score were established using the Pearson correlation coefficient. The statistical analyses were carried out based on R (version 4.0.5).

## Results

### Ferroptosis genes were correlated with prognosis in human GBMs

Using univariate regression analysis, we screened 14 ferroptosis genes associated with OS in patients with GBM. According to the optimal threshold of the level of these genes in the TCGA-GBM, the cases were divided into two groups with high and low expression. The KM curve showed significant prognostic differences between the high and low gene expression groups (*p*-value < 0.05). The PCA algorithm was applied to determine the PCA score of each TCGA-GBM individual based on these 14 genes. The results showed that a high score has a positive relationship with prognosis (*p* = 0.002, **Figure 1B**).

### Identification of DEGs between the high-score and low-score groups

Up to 231 DEGs were identified, among which 183 were upregulated and 48 were downregulated in the high-score group. To further explore the biological role of these ferroptosis-related genes in GBM, we performed GO and KEGG analyses on these DEGs. The GO result reflected that the GO annotations of DEGs can be classified into three categories: biological processes (BPs), cell components (CCs), and molecular functions (MFs). The terms were arranged based on the false discovery rate (FDR) value, and further screening was performed when FDR <0.05. Screening showed that the DEGs were enriched in processes of regulation of long-term neuronal synaptic plasticity, proteasome-mediated ubiquitin-dependent protein degradation, vesicle-mediated transport in synapses, and proteasomal protein catabolic processes (**Figures S1A, B**). The signal pathway results were ranked based on FDR value and were screened under the condition of FDR <0.05. The KEGG result reflected that the DEGs were mainly enriched in endocytosis, Alzheimer’s disease, and pathways of neurodegeneration in multiple diseases (**Figures S1C, D**).

### Tumor ferroptosis-related classification according to the identified DEGs

**Figure 2:**
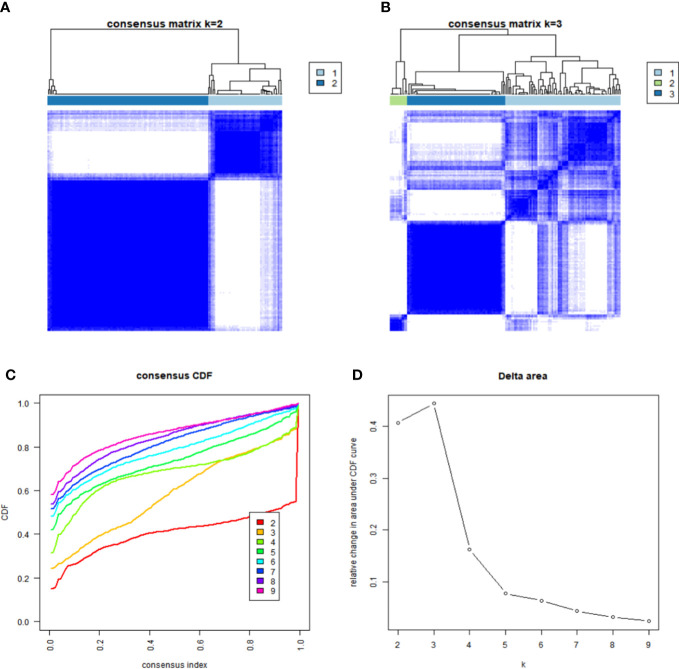
Identification of GBM ferroptosis-related subgroups. **(A, B)** Heatmap of the samples under the condition of consensus *k* = 2 **(A)** and *k* = 3 **(B)**. **(C)** Consensus clustering cumulative distribution function (CDF) for *k* = 2 to 9. **(D)** Relative change in the area under the CDF curve for *k* = 2 to 9.

**Figure 3:**
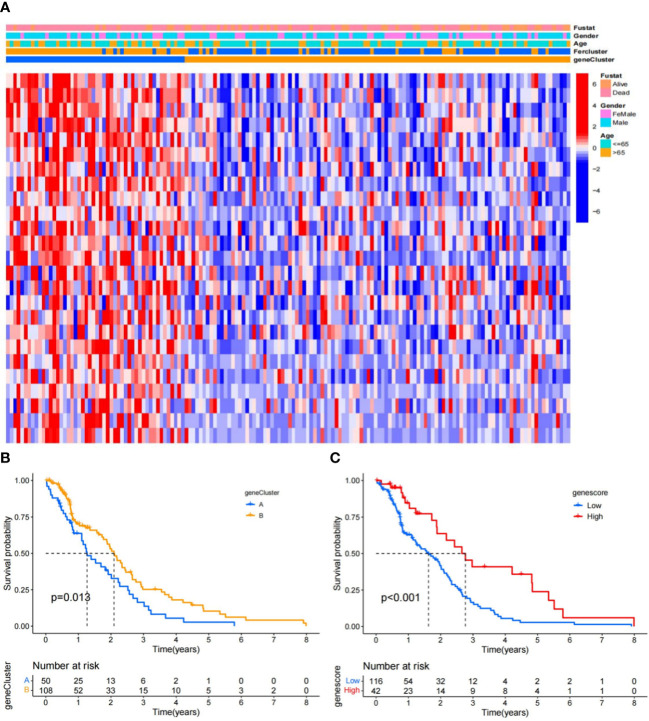
Ferroptosis signatures. **(A)** Unsupervised clustering of overlapping ferroptosis-related genes in the TCGA-GBM cohorts to classify cases, termed as gene clusters A and B, respectively. The gene clusters, PCA scores, and age were applied as patient annotations. **(B)** KM curves of the gene clusters. **(C)** KM curves of the gene scores in the TCGA-GBM.

To study the association of the expression level and GBM subtypes of the 231 ferroptosis-related DEGs, we conducted univariate Cox regression and consensus clustering test with all GBM cases in the TCGA. When the clustering variable (*k*) increased from 2 to 10, it can be found that when *k* = 2, there appeared the highest intragroup associations and the lowest intergroup associations, which reflected that the 158 GBM cases can be perfectly divided into two groups by the 25 prognostic DEGs (**Figures 2A–D**). The clinical features, such as PCA score (high score or low score), sex (male or female), age (≤65 or >65 years), and survival condition, were listed in a heatmap (**Figure 3A**), and a few differences in sex and age between the two groups were found. The OS rates were also compared, and we found significant differences (*p* = 0.013, **Figure 3B**). To study the prognostic effects of these genes, the PCA algorithm was also applied to determine the PCA gene score of each individual, and cases were divided into two groups. The KM curves showed that the high-score group had obviously a better prognosis (*p* < 0.001, **Figure 3C**).

### Comparison of the immune activity

Based on the above analysis, this study used ssGSEA to compare the enrichment fraction of 16 types of immune cells and the activity difference of 13 immune-related pathways among groups in detail. The comparative analysis results show that in the TCGA queue (**Figures S2A, B**), the immune cell infiltration of the subgroup with high gene score is also higher, and the corresponding immune infiltration is mainly related to activated CD8^+^T, CD4^+^T, and Treg cells. The comparison results also showed that the infiltration of neutrophils and NK and Th cells in the high-score subgroup was more obvious.

**Figure 4:**
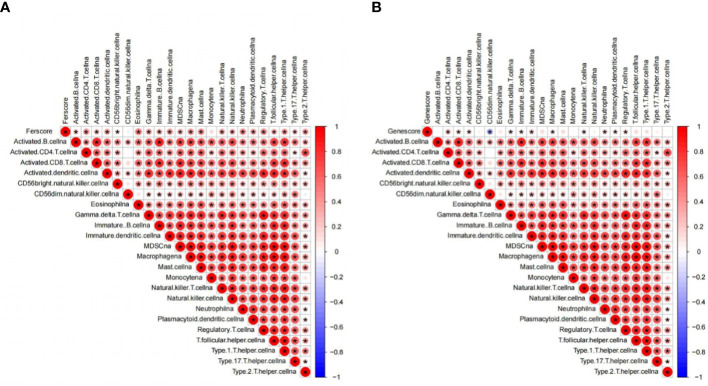
The association of immune cells and PCA score **(A)** and PCA gene score **(B)**.

Some immune pathways, such as the TGF beta pathway, NK cell-mediated cytotoxicity pathway, JAK–STAT pathway, Toll-like receptor pathway, and B-cell receptor pathway, showed higher activity in the high-score group (**Figure S2C**). Moreover, amino sugar/nucleotide sugar/starch/sucrose metabolism and other glycan degradation pathways have higher activity in the high-gene score group, while taurine/hypotaurine-glycine/serine/threonine metabolism, calcium signaling, and neuroactive ligand–receptor interactions have lower activity in the high-gene score group (**Figure S2D**). According to the comparative analysis results, the activity of the sucrose degradation pathway in the high-gene score group is significantly higher than that in the other group, but the corresponding calcium signaling and neural activity are relatively low (**Figure S2D**). The cause is not very clear, and it needs to be analyzed in detail to provide support for clarifying the pathological correlation between ferroptosis and this disease, so as to better meet the requirements related to treatment. Correlation analysis showed a significant positive correlation between immune cells in GBM, and almost all immune cells have a positive association with the PCA score and PCA gene score (**Figures 4A, B**).

### Verification of potential biomarker expression

**Figure 5:**
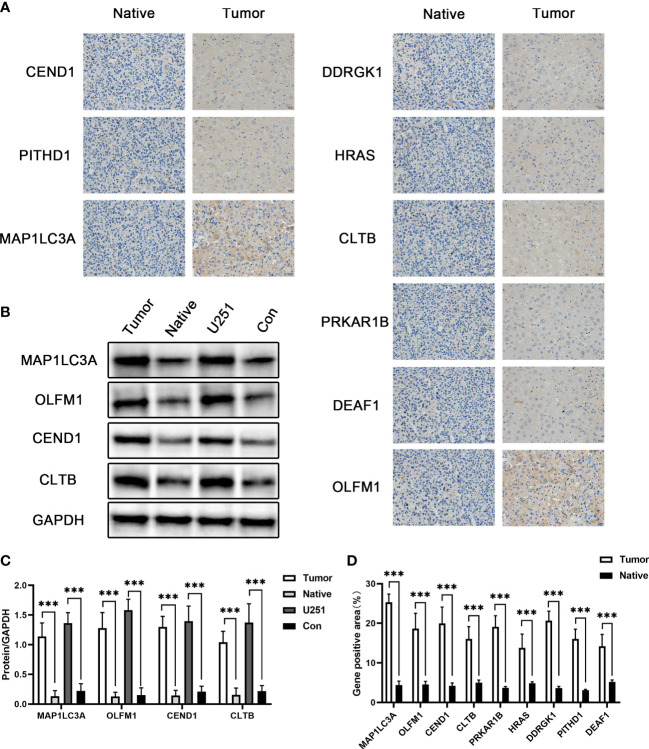
The expression of potential biomarkers. **(A)** Immunohistochemical detection of MAP1LC3A, OLFM1, CEND1, CLTB, PRKAR1B, HRAS, DDRGK1, PITHD1, and DEAF1 gene expression. **(B)** Western blot detection of the MAP1LC3A, OLFM1, CEND1, and CLTB protein expression levels. **(C)** Quantitative results of Western blotting. **(D)** Quantitative results of immunohistochemistry. *** means p value less than 0.001.

In order to further verify the role of the 25 differential genes we screened earlier in glioma, we detected the expression of the above genes in glioma glial tissue and glioma paracancerous glial tissue by immunohistochemistry, and the expression of the above genes in glioma glial tissue was significantly increased. Next, we detected four genes with differential expression greater than 10-fold by Western blot at the cellular and tissue levels, namely, *MAP1LC3A*, *OLFM1*, *CEND1*, and *CLTB*. The results showed that the expression levels of the above proteins were significantly upregulated relative to both glioma paracancerous glial cells and glioma paracancerous glial tissue. This further validates the results of our previous analysis (**Figure 5**).

## Discussion

Malignant glioma is a fatal tumor, and many scholars found that ferroptosis is closely associated with glioma (31–34). Ferroptosis is closely related to iron-mediated oxidative stress, cellular metabolism, and autophagy (35). Ferroptosis is very important in the development of glioma, while the related mechanism is still unclear. One study on the role and mechanism of ferroptosis in glioma can provide a better understanding of the pathogenesis of glioma and help to find new targets for the comprehensive treatment of glioma. Therefore, this study mainly aimed to identify DEGs associated with ferroptosis to offer a reliable theoretical basis for glioma therapy and construct a prognosis score for individual patients to better manage glioma treatment.

Ferroptosis inducers have high application value in tumor treatment (36). In cancer treatment, drug resistance is the key issue to be addressed. The comparative analysis showed that the process of cell death and apoptosis was significantly different in patients with iron deficiency syndrome. This drug can provide support in dealing with the drug resistance of tumors (37). Studies have found that activation of the ferroptosis pathway will promote the death of cancer cells, which can solve the problem of drug resistance, and also improve the sensitivity of cancer cells to drugs. The combined use of ferroptosis inducers and chemotherapy may achieve a synergistic reaction, thus improving sensitivity to chemotherapy. There is compelling evidence that glutathione peroxidase 4 inhibitors enhance the lethality of drug-resistant cells *via* ferroptosis which may help prevent acquired drug resistance in tumors (38). In addition, cisplatin in combination with erastin has been shown to significantly increase antitumor activity, suggesting that ferroptosis is critical in tumor therapy (39). It can improve the sensitivity of cancer cells to chemotherapy drugs, which is conducive to reducing the dosage and adverse drug reactions and is of great significance for improving the therapeutic effect. Autophagy inhibition in GBMs can enhance the sensitivity of GBM stem cells to treatment by causing ferroptosis (20). The combination of TMZ and erastin can provide effective therapy. Therefore, research on the treatment of ferroptosis may provide a new therapy approach for those who are resistant to traditional radiotherapy and chemotherapy or those who have not responded to immunotherapy.

In this study, we found that some ferroptosis genes could predict the OS of patients. Based on these genes, the PCA method was applied to determine the individual scores of patients and divide them into high- and low-score groups. Survival analysis reflected significant differences in OS between the two groups. Subsequently, 231 DEGs were identified between different score groups, and biological function analysis showed that these genes were mainly involved in the regulation of long-term neuronal synaptic plasticity, proteasome-mediated ubiquitin-dependent protein degradation, vesicle-mediated transport in synapses, proteasomal protein catabolic processes, etc. There were significant differences in immune activity between the two groups, suggesting that patients in the high-score group were in a state of immune activation. We further screened 231 DEGs for 25 prognostic DEGs and then used these genes for unsupervised classification. The OS rates were also compared and significant differences were found. In addition, the PCA algorithm was also applied to determine the PCA gene score of each patient, and cases were divided into high- and low-gene score groups. The survival analysis showed that the high-gene score group exhibited obviously a better prognosis. The high-gene score group had higher immune cell infiltration. Correlation analysis showed that the immune cells were positively associated with the PCA score and PCA gene score.

This study showed that GBM samples can be divided into two different groups using the PCA algorithm based on ferroptosis genes, and the low-score group has a worse prognosis. Subsequently, we identified 231 DEGs between different score groups, and 25 ferroptosis-related DEGs were identified based on Cox regression. The GBM samples were divided into two different clusters by an unsupervised clustering method. There were significant prognostic differences and immunoactivity between the two groups. In terms of the 25 prognostic DEGs, we further calculated individual risk scores using the PCA algorithm, which can be used to effectively predict the prognosis of a patient with glioma. Moreover, this research offers a novel understanding of the occurrence of ferroptosis in the progress of glioma and provides a valuable idea for the development of ferroptosis inducers for glioma therapy. Given that these partial results were generated using the public RNA-seq technique, further studies are needed to investigate the prognostic function of these 25 gene signatures.

## Data availability statement

The original contributions presented in the study are included in the article/**Supplementary Material**. Further inquiries can be directed to the corresponding author.

## Author contributions

All authors designed the study, analyzed the data, and drafted the manuscript. All authors contributed to the article and approved the submitted version.
