# Supplementary material for: A novel 25-ferroptosis-related gene signature for the prognosis of gliomas
Source: Front Oncol. 2023 Apr 20;13:1128278. doi: 10.3389/fonc.2023.1128278 (PMC10157171; doi:10.3389/fonc.2023.1128278)

**Figure S1. Functional analysis based on the DEGs between the high-score and low-score groups in the TCGA-GBM cohort.** (A, B) Bubble graph (A) and barplot graph (B) for GO enrichment; (C, D) Bubble graph (C) and barplot graph (D) for KEGG pathways. For Bubble graph, the bigger bubble means the more genes enriched, and the increasing depth of red means the differences were more obvious; q-value: the adjusted p-value. For barplot graph, the longer bar means the more genes enriched, and the increasing depth of red means the differences were more obvious.


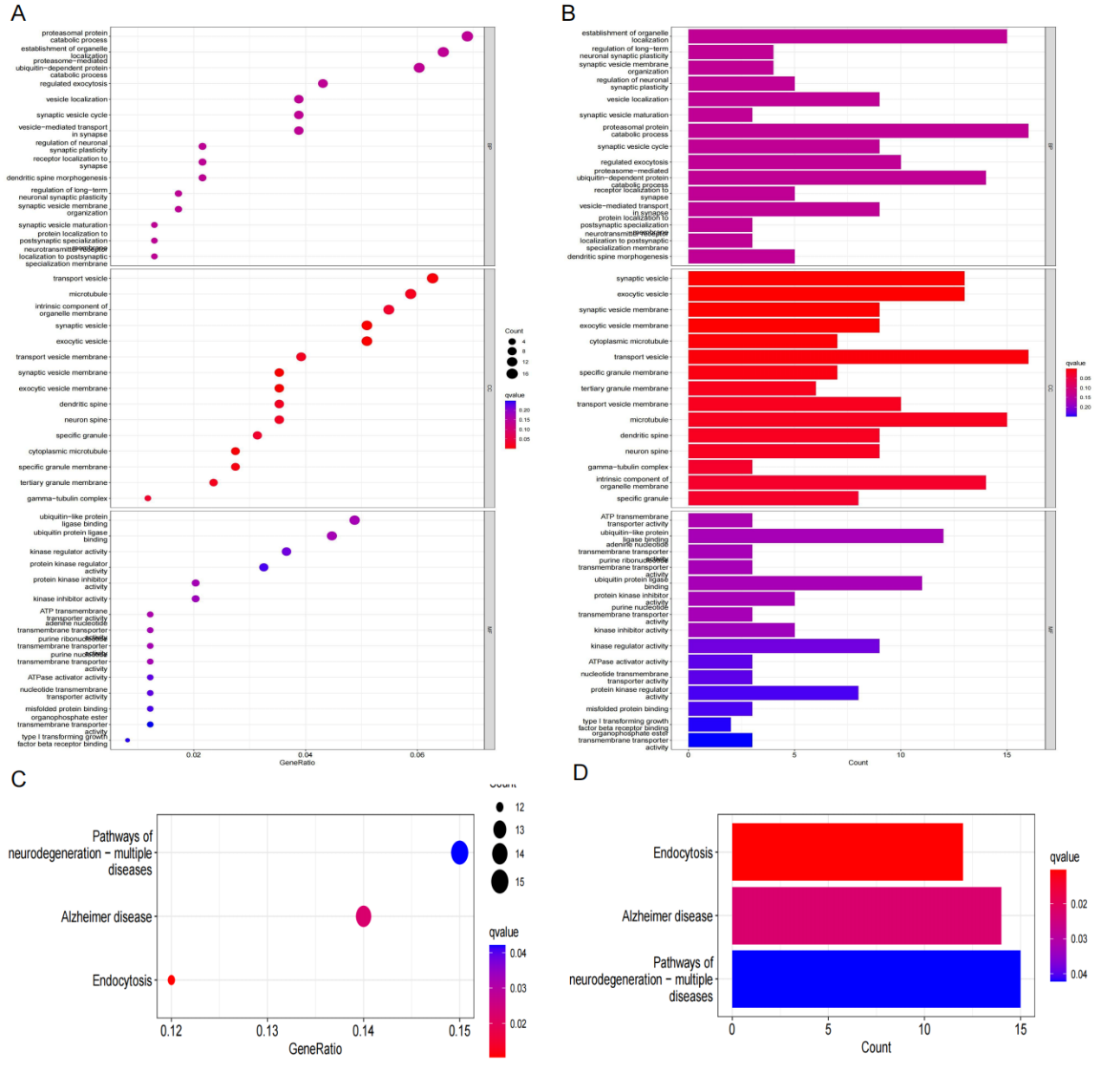


**Figure S2. The results of Single-Sample Gene Set Enrichment Analysis.** (A, B) The fractions of 16 types of infiltrating immune cells in samples from 2 scorecluster groups (A: high-score and low-score groups) and 2 genecluster (B: high-genescore and low-genescore groups); (C, D) The heat maps of pathways in samples from 2 scorecluster groups (C: high-score and low-score groups) and 2 genecluster (D: high-genescore and low-genescore groups).


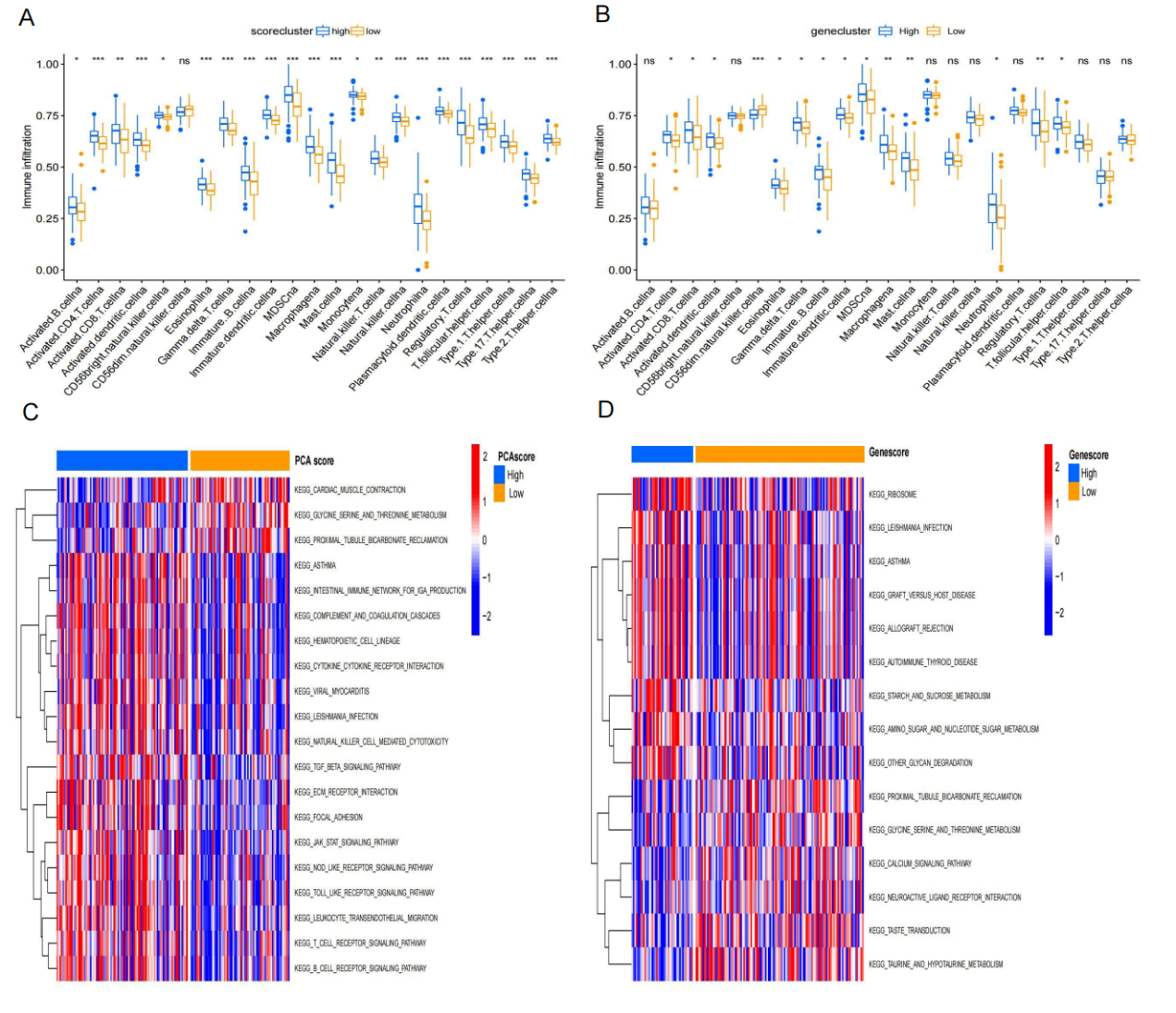

Supplement: Supplementary file 1 [file DataSheet_1.docx]
